# *Artemisia arborescens* and *Artemisia inculta* from Crete; Secondary Metabolites, Trace Metals and In Vitro Antioxidant Activities

**DOI:** 10.3390/life13061416

**Published:** 2023-06-19

**Authors:** Dimitra Z. Lantzouraki, Charalampia Amerikanou, Sotirios Karavoltsos, Vasiliki Kafourou, Aikaterini Sakellari, Dimitra Tagkouli, Panagiotis Zoumpoulakis, Dimitris P. Makris, Nick Kalogeropoulos, Andriana C. Kaliora

**Affiliations:** 1 Institute of Chemical Biology, National Hellenic Research Foundation, 48 Vas. Constantinou Ave., 11635 Athens, Greece; dlantzouraki@gmail.com; 2 Department of Nutrition and Dietetics, School of Health Science and Education, Harokopio University, 70 El. Venizelou Ave., 17676 Athens, Greece; amerikanou@windowslive.com (C.A.);; 3 Laboratory of Environmental Chemistry, Department of Chemistry, National and Kapodistrian University of Athens, 15784 Athens, Greece; skarav@chem.uoa.gr (S.K.);; 4 Department of Food Science and Technology, University of West Attica, Ag. Spyridonos, 12243 Egaleo, Greece; 5 Department of Food Science & Nutrition, School of Agricultural Sciences, University of Thessaly, N. Temponera Street, 43100 Karditsa, Greece

**Keywords:** *Artemisia arborescens*, *Artemisia inculta*, phenolic compounds, terpenes, trace metals, antioxidant capacity

## Abstract

Background: Currently, the use of medicinal plants has increased. *Artemisia* species have been used in several applications, including medicinal use and uses in cosmetics, foods and beverages. *Artemisia arborescens* L. and *Artemisia inculta* are part of the Mediterranean diet in the form of aqueous infusions. Herein, we aimed to compare the secondary metabolites of the decoctions and two different extracts (methanolic and aqueous-glycerolic) of these two species, as well as their antioxidant capacity and trace metal levels. Methods: Total phenolic, total flavonoid, total terpenes, total hydroxycinnamate, total flavonol, total anthocyanin contents and antioxidant/antiradical activity were determined, and GC/MS analysis was applied to identify and quantify phenolics and terpenoids. Trace metals were quantified with ICP-MS. Results: Aqueous-glycerolic extracts demonstrated higher levels of total secondary metabolites, greater antioxidant potential and higher terpenoid levels than decoctions and methanolic extracts. Subsequently, the aqueous-glycerolic extract of a particularly high phenolic content was further analyzed applying targeted LC-MS/MS as the most appropriate analytic tool for the determination of the phenolic profile. Overall, twenty-two metabolites were identified. The potential contribution of infusions consumption to metal intake was additionally evaluated, and did not exceed the recommended daily intake. Conclusions: Our results support the use of these two species in several food, cosmetic or pharmaceutical applications.

## 1. Introduction

To meet their treatment needs, nowadays most people rely on traditional herbs, and 60% of medicines in pharmacies are derived from medicinal plants [1]. The use of medicinal plants and their associated formulations is becoming more common throughout the world due to the fact that they are available, safe, effective and the subject of valuable traditional knowledge that can be used to prevent and treat a variety of diseases [2,3,4]. On the other hand, several high-priced medications that are routinely used have unpredictable and serious side effects. Thus, as a consequence of the increasing demand for new therapeutic strategies worldwide, it is crucial to investigate botanical plants in terms of their potential for safe treatments methods. For example, it has been established that bioactive phytochemical compounds detected in several botanic species could be used to prevent and treat diseases linked with oxidative stress, such as diabetes, cardiovascular diseases, different forms of cancer, rheumatoid arthritis or Alzheimer’s disease [5].

*Artemisia* is a plant genus of the Asteraceae family with hundreds of species, mainly found in the drier climates of the Northern Hemisphere, with several culinary, beverage, aromatic and industrial uses [6]. For instance, on the island of Crete, Greece, *Artemisia arborescens* L. or *Arboreus absinth* and *Artemisia inculta Delile* constitute part of the Mediterranean diet in the form of aqueous infusions. *Artemisia* species are of great importance in traditional medicine, mentioned even in ancient sources for the treatment of fever, dysentery and hemorrhoids, as an antispasmodic or for calming of children [7]. In recent years, *Artemisia* species have attracted considerable research interest because of their chemical composition and biological activities [8]. The 2015 Nobel Prize in Physiology or Medicine was awarded to Professor Youyou Tu for her key contribution to the discovery of artemisinin, a new class of antimalarial drugs that have saved millions of lives and represents one of the significant contributions of China to global health. Different classes of secondary metabolites have been detected among the 260 *Artemisia* species, including lignans, sesquiterpenoids, flavonoids, coumarins, glycosides, caffeoylquinic acids, sterols and polyacetylenes [9,10]. Additionally, different species of *Artemisia* exhibit neuroprotective, antidepressant, cytotoxic, digestive and antimicrobial activities [11,12,13,14] as well as nephroprotective [15] or hepatoprotective [16] properties. Added to the above, the use of *Artemisia* species in cosmetic products has increased significantly, mostly due to their antibacterial or antioxidant properties [11].

The aforementioned significant health benefits of *Artemisia* species promote an increase in the consumption, and several other uses, of this plant. However, particular attention should be paid to the metal content of the plant material used, since certain metals’ gradual accumulation in vital organs, combined with their incomplete excretion from the human organism, poses a serious health risk [17,18]. Among the metals most frequently examined in the literature, also studied here were Co, Fe, Mn and Zn, representing essential nutrients; Cr, Cu and Ni, which are essential, albeit exerting toxicity only at elevated concentrations; and Cd and Pb, being exclusively toxic with no beneficial properties even at low levels.

The research of *Artemisia* species from the island of Crete, Greece, the southernmost point of Europe, is limited. To the best of our knowledge, this is the first study that evaluates the main phytochemical compounds of *A. arborescens* and *A. inculta* from Crete following different extraction methodologies, as well as their antioxidant activities and trace metals. Additionally, another novelty lies in the proposed aqueous-glycerolic method as it yields products with high secondary metabolite contents, antioxidant capacity and acceptable levels of trace elements in terms of toxicity.

## 2. Materials and Methods

### 2.1. Chemicals, Standards and Solvents

Ferric chloride hexahydrate (FeCl_3_ 6H_2_O) of analytical grade was supplied from Acros Organics (Morris Plains, NJ, USA), and aluminum chloride (AlCl_3_) from Fisher Scientific (Princeton, NJ, USA). Gallic acid, Folin–Ciocalteu’s phenol reagent, rutin (quercetin 3-O-rutinoside), ascorbic acid, 2,2′-diphenyl-1-picrylhydrazyl (DPPH●) free radical, p-(dimethylamino)-cinnamaldehyde (DMAC), (+)-catechin, Trolox 6-hydroxy-2,5,7,8-tetramethylchroman-2-carboxylic acid and 2,4,6-tripyridyl-s-triazine (TPTZ) were purchased from Sigma Chemical Co. (St. Louis, MO, USA). Standard phenolic compounds—namely, 3,4,5-trihydroxybenzoic acid, trans-4-hydroxycinnamic acid, 3,4-dihydroxycinnamic acid, vanillin and quercetin—were purchased from Alfa Aesar (Karlsruhe, Germany), while (±)-naringenin and (±)-catechin were obtained from Sigma-Aldrich (Steinheim, Germany). Cinnamic acid, 4-hydroxybenzoic acid, nitric acid 65% supra pure and hydrogen peroxide 30% supra pure were purchased from Merck KGaA (Darmstadt, Germany), 2,6-di-tert-butyl-4-methylphenol was purchased from Acros Organics (Geel, Belgium) and 2-(4-hydroxyphenyl) ethanol was purchased from Fluka Analytical (Merck KGaA, Darmstadt, Germany). All solvents used were of GC, HPLC or MS grade and were purchased from Sigma Aldrich Co. (Gillingham, UK), Fisher Chemical (Loughborough, UK) and Merck KGaA (Darmstadt, Germany). Glycerol purchased from Oleon Corporate M&S (Ertvelde, Belgium) and used for extractions was 99 % pure. Formic acid of MS grade was purchased from LGC Standards (Wesel, Germany).

### 2.2. Sampling and Preparation

The plant material used in this study consisted of the aerial parts of *A. inculta* Delile and *A. arborescens*, which were provided by the Mediterranean Plant Conservation Unit, The Mediterranean Agronomic Institute of Chania (M.A.I.Ch., Chania, Crete, Greece), where voucher specimens were deposited (*A. inculta*: 9493 MAIC; *A. arborescens*: 9504 MAIC). The aerial parts, composed of foliage and stems, of *A. arborescens* and *A. inculta* were collected from the Almyrida area (Apokoronas region of Chania regional unit, Crete, Greece) and the island of Gavdos (regional unit of Chania, Crete, Greece), respectively. In particular, the different samples from the experts of M.A.I.Ch. were collected from fully grown shrubs during in October 2014 and October 2015 (mean values of temperature and daily rainfall in Almyrida area were 19.9 °C, 4.23 mm and 20.8 °C, 3.30 mm in October 2014 and October 2015, respectively; the corresponding data for Gavdos island were 16.0 °C, 2.56 mm in October 2014 and 16.3 °C, 3.11 mm in October 2015).

Stems of plant samples were discarded, while flowering tops and leaves were carefully washed in cold distilled water, drained and left to dry at room temperature in a dry dark chamber for 7 days. Dried samples were grounded to a fine powder in a mechanical grinder and stored in the dark at 4 °C until their further use within 4 months post-collection.

### 2.3. Preparation of Samples

Each sample of *A. arborescens* and *A. inculta* obtained from the two samplings was homogenized separately. Methodologies followed for the preparation of herbal decoctions or methanolic and aqueous-glycerolic extracts were identical for the two species, while each procedure was carried out in triplicate. All *Artemisia* samples prepared as indicated below were stored at −80 °C in darkness until analysis.

#### 2.3.1. Decoctions

Decoctions were prepared by adding 3 g of dried herb to 200 mL [1:67 (*w*/*v*) material to solvent ratio] of bottled natural mineral water in a glass (Pyrex) boiling pot. The mixture was placed on a preheated heating plate, left at boiling temperature for 3 min, then at room temperature for 2 min, and finally filtered by a Buchner funnel under vacuum. An appropriate amount of water was added to maintain the final volume of 200 mL. Decoctions were freeze-dried for 120 h in a Cryodos freeze dryer (Telstar Industrial, Barcelona, Spain) and the dry residue was weighed. Furthermore, the residue of total salts per volume of the mineral water used was determined after freeze-drying for the correction of extractable yield values. Freeze-dried decoctions were stored at −80 °C in darkness and appropriately diluted prior to analysis (3 g dry residue of decoction per 200 mL distilled water).

#### 2.3.2. Herbal Extracts

For the methanolic extracts, a classical extraction procedure was performed with a 1:100 (*w*/*v*) material to solvent ratio as follows. Approximately 0.5 g of dried herb was macerated in 50 mL of methanol, and the mixture was left in darkness under constant stirring at room temperature for 24 h. The crude extracts were then centrifuged at 3600 rpm for 10 min, and the supernatants were collected and evaporated to dryness using a rotary evaporator at 40 °C. Each dry residue was separately redissolved in 10 mL of methanol and the solvent was evaporated to dryness once more using a centrifugal concentrator (Speed Vac, Labconco Corporation, Kansas City, MO, USA) at 40 °C. Finally, the dried extracts were redissolved in 2 mL of methanol using an ultrasonic bath. The concentrated samples were preserved in the dark at −80 °C and diluted to a final concentration of 1 g of extract dry residue per 100 mL of methanol for further analysis.

The glycerol–water extracts were prepared as described by Shehata et al. [19] with minor modifications. One (1) gram of dried plant sample was mixed with 125 mL of glycerol–water 9:1 (*w*/*v*) mixture, and the extraction took place on a magnetic stirrer hot plate at 80 (±1) °C, under continuous stirring for 160 min. The extracts were then cooled in a water bath at room temperature, centrifuged at 3500 rpm for 30 min, and the supernatants were collected for further analysis.

### 2.4. Determination of Total Phenolic Content

Total phenolic content (TPC) of each sample preparation was determined by applying a micro method of Folin–Ciocalteu’s colorimetric assay, based on the procedure described by Karakashov et al. [20]. Briefly, in a 1.5 mL tube, 20 μL of sample, standard solution or blank was added to 780 μL of distilled water and 50 μL of Folin–Ciocalteu reagent, mixed thoroughly and then allowed to stand for 1 min. Subsequently, 150 μL of saturated (20% *w*/*v*) aqueous sodium carbonate solution was added, and the mixture was vortexed and allowed to stand at room temperature in darkness for 60 min. The samples were transferred to a 96-well plate, and the absorbance was measured at 750 nm using an ELISA microplate reader (Power Wave XS2, Microplate Spectrophotometer, BioTekInstruments, Winooski, VT, USA). The TPC was expressed as mg of caffeic acid equivalents (CAE) per gram of dried *Artemisia* sp. using a standard curve within a range of 40–1000 mg·L^−1^ caffeic acid in assay solution (y = 0.0007x − 0.0129, R^2^ = 0.998).

### 2.5. Determination of Total Flavonoid Content (TFC)

For estimating the total flavonoid content (TFC) of decoctions or extract samples of the two *Artemisia* species, a previously published protocol was applied with some modifications [21]. In detail, an aliquot of 25 μL of sample was mixed with 30 μL of sodium nitrite (NaNO_2_) aqueous solution 5 % (*w*/*v*), and the derived solution was incubated in 300 μL of ethanol–water 1:1 (*v*/*v*) for 5 min at room temperature. Afterwards, 150 μL of aluminum chloride hexahydrate solution (AlCl_3_·6H_2_O) 2% (*w*/*v*) in water was added and allowed to stand at room temperature for 5 min. After the addition of 200 μL of sodium hydroxide (NaOH) 1 M aqueous solution, the mixture was adjusted to a final volume of 1 mL with ethanol–water 1:1 (*v*/*v*). The absorbance was measured at 510 nm using a 96-well plate and an ELISA microplate reader, while the total flavonoid concentration was expressed as mg catechin equivalents (CE) per gram of dried *Artemisia* species. The range of the concentrations for catechin was 20–1000 mg·L^−1^ in assay solution (y = 0.0003x + 0.0048, R^2^ = 0.998). 

### 2.6. Determination of Phenolic Classes

The methodology employed by Galanakis et al. [22] was performed to determine different phenolic classes in the extracts and decoctions of *Artemisia* sp., namely, hydroxycinnamates, flavonols and anthocyanins. In short, 1 mL of each sample and 1 mL of aqueous ethanol (95% *v*/*v*) containing 0.1% (*v*/*v*) hydrochloric acid were mixed to a final volume of 10 mL with 2% (*v*/*v*) hydrochloric acid. The absorbance of the mixture was measured at 320, 360 and 520 nm to determine total hydroxycinnamate content (THCC) as mg of caffeic acid equivalents (CAE) per gram of dried *Artemisia* sp., total flavonol content (TFnolC) as mg of quercetin equivalents (QE) per gram of dried plant and total anthocyanin content (TAC) as μg of cyanidin equivalents (CNE) per gram of dried plant, respectively. Concentration ranges and equations of the corresponding standard curves of the above-mentioned determinations were as follows: caffeic acid, 5–20 mg·L^−1^ of assay solution, y = 0.0686x − 0.0100 (R^2^ = 0.998); quercetin, 5–20 mg·L^−1^ of assay solution, y = 0.0444x − 0.0290 (R^2^ = 0.999); cyanidin chloride, 40–300 μg·L^−1^ of assay solution, y = 0.0009x − 0.0153 (R^2^ = 0.990).

### 2.7. Determination of Total Terpenes

A colorimetric assay method based on Fan and He [23] was used to estimate the content of total terpenic compounds (TTC). For each sample preparation, 200 μL were evaporated to dryness in a boiling water bath. The dry residue was re-diluted with 0.3 mL (5% *w*/*v*) vanillin in glacial acetic acid and 1 mL of perchloric acid solution. The mixture was heated for 45 min at 60 °C and then cooled in an ice-water bath to ambient temperature. The absorbance of assay solutions was measured at 548 nm following the addition of 5 mL glacial acetic acid. Ursolic acid was used as the standard compound within a range of 3–30 mg·L^−1^ of the assay solution (y = 0.0298x − 0.0664, R^2^ = 0.988). The TTC of extracts and decoctions was expressed as mg of ursolic acid equivalents (UAE) per gram of dried *Artemisia* plant.

### 2.8. Assessment of Antioxidant Activity

The antioxidant activity was assessed by measuring the radical-scavenging activity and reducing antioxidant potential of *Artemisia* decoctions and extracts.

The antiradical power of tested *Artemisia* preparations was assessed as described in a previous study [24]. The DPPH assay provides an evaluation of the samples’ potency to scavenge the 2,2′-diphenyl-1-picrylhydrazyl free radical, which was depicted as the concentration of Trolox equivalents (TE) per gram of dry herb, using a standard curve ranging from 0.050 to 1.2 mM of Trolox (y = 0.31504x + 0.00161, R^2^ = 0.993). The absorbance was recorded at 515 nm twice, i.e., at 5 and 30 min, where the absorbance was stabilized at a minimum value. 

The antioxidant power of *Artemisia* decoctions and extracts was evaluated based on the reduction of iron from ferric to ferrous form when being complexed with 2,4,6-tris(2-pyridyl)-s-triazine (TPTZ). Ferric Reducing/Antioxidant Power (FRAP) assay was carried out according to a previously published work [25]. For the construction of the standard curve (y = 0.0.83601x + 0.04754, R^2^ = 0.999), 10–500 μM of standard solutions of L-ascorbic acid were prepared. The absorbance for samples, blanks and standards was measured until stabilization to a peak value at 620 nm, and the results were expressed as mg of L-ascorbic acid equivalents (AAE) per gram of dry herb.

In addition, inhibition of copper-induced lipid oxidation in total serum solubilized in phosphate buffer saline (PBS), using lag time as a criterion for antioxidative potency, was evaluated as a more biologically relevant assay to assess the antioxidant activity of the *Artemisia* samples. Venous blood was collected under sterile conditions from healthy humans, and serum was obtained after centrifugation at 3000 rpm at 4 °C for 10 min directly after collection. The study of the kinetics of copper-induced oxidation in 12-fold diluted serum was performed by monitoring the absorbance of lipid oxidation products at 245 nm using an ELISA reader (PowerWaveXS2, Microplate Spectrophotometer, BioTek, Winooski, VT, USA). At time point 0, CuSO_4_ was added in the serum (20 μL) to a final concentration of 10^−5^ M in PBS. Copper-induced oxidation of lipids in serum leads to the formation of conjugated dienic hydroperoxides that absorb at 245 nm. The kinetics of oxidation was analyzed in terms of the lag time prior to oxidation and was expressed in seconds.

### 2.9. GC/MS Analysis of Phenolic Compounds and Terpenoids

Gas chromatography/mass spectrometry (GC/MS) analysis of phenolic and terpenic compounds was performed. An Agilent (Wallborn, Germany) HP series GC 6890N coupled with a HP 5973 MS detector (EI, 70 eV), split–splitless injector and an HP 7683 autosampler were used for the determination of phenolic and terpenic compounds of *Artemisia* decoctions and methanolic or hydroglycerolic extracts. An aliquot (1 μL) of the silylated samples was injected into the gas chromatograph at a split ratio of 1:20. Separations were achieved on a HP-5 MS capillary column (30 m × 0.25 mm × 250 μm), employing high purity helium at 0.6 mL/min as the carrier gas. The injector and transfer line temperatures were kept at 250 and 300 °C, respectively, and the oven temperature was kept initially at 70 °C for 5 min, then raised to 70–130 °C at 15 °C /min, then 130–160 °C at 4 °C/min, kept at 160 °C for 15 min and finally raised to 160–300 °C at 10 °C/min and kept at 300 °C for 15 min. 

A selective ion monitoring (SIM) GC/MS method was applied for the detection of 17 phenolic compounds, 1 stilbene, 4 terpenic compounds and the internal standard based on the ±0.05 RT presence of target and qualifier ions of commercial standard compounds at the predetermined ratios. The target and qualifier ions used for the identification of compounds under concern are presented in Table S1. Identification of chromatographic peaks was made by comparing the retention times and ratios of two or three fragment ions of each phenolic or terpenic compound with those of commercial reference standards [26]. Quantification was carried out by constructing reference curves for each compound, based on a series of 9 standard mixtures of the phenolic and terpenic compounds containing the same quantity of internal standard as that of samples. Serving as the internal standard was 3-(4-hydroxyphenyl)-1-propanol.

### 2.10. Targeted LC-MS/MS Profiling of Phenolic Compounds in Glycerolic Extracts

Liquid chromatography–mass spectrometry (LC-MS) was employed for further investigation of phenolic compounds in the aqueous-glycerolic extracts of *Artemisia* sp. samples, as previously described [24,27]. HPLC-PDA-ESI-MS/MS paired with an in-house multiple reaction monitoring (MRM) spectral library was employed. Phenolic compound separation was carried out using a Thermo Scientific Surveyor Plus HPLC-PDA-ESI-MS/MS system (San José, CA, USA). The platform comprises a Thermo Scientific Surveyor HPLC Pump Plus, a Thermo Scientific Surveyor Autosampler Plus Lite and an LCQ FLEET mass spectrometer equipped with an Electrospray Ionization (ESI) Probe and an Ion Trap analyzer. Data were processed using the Xcalibur software program (version 2.1).

Prior to LC-MS analysis, the extracts were diluted as 1:2 (*v*/*v*) with a mixture of MeOH-H_2_O 7:3 (*v*/*v*). The chromatographic separation of phenolics was carried out using a Finnigan Surveyor system and a Hypersil Gold Column (3 mm, 2.1 × 100 mm, Thermo, Palo Alto, Santa Clara, CA, USA) protected with a security guard cartridge (Hypersil Gold, 3 mm, 10 × 2.1 mm i.d.). The gradient mobile phase consisted of solvent A [water—0.5% (*v*/*v*) formic acid] and solvent B (acetonitrile). The flow rate was 0.3 mL·min^–1^ and the injection volume was 5.0 μL. The gradient elution program was initially 5% B, linear 5–9% B at 4 min, linear 9–15% B at 8 min, linear 15–18% B at 11 min, held constant for 1 min, linear 18–50% B at 15 min, held constant for 2 min, purging with 100% B during 6 min and re-equilibration of the column for 10 min.

Mass spectrometric analysis of sample solutions was operated in negative electrospray ionization (ESI) mode, and different collision energies were applied for tandem MS analysis. Mass spectrometer parameters for negative ion mode were as follows: source voltage, 4.0 kV; capillary voltage, −18 V; capillary temperature, 300 °C; sheath gas flow, 50 (arbitrary units); sweep gas flow, 20 (arbitrary units); full max ion time, 300 ms; and full micro scans, 3. MRM experiments were performed by specifying the deprotonated parent ion of each targeted compound for MS/MS fragmentation and the fragment ions were recorded. For the identification of each compound, the parent ion from the negative ionization mode as well as the characteristic fragments deriving from the fragmentation were used. Data-dependent scans for MS/MS analyses were carried out with the following conditions: collision energies, 15, 25, 30, 35 (arbitrary units); width, 1.00; repeat count, 2; repeat duration, 0.5 min; exclusion size list, 25; exclusion duration, 1.00 min; exclusion mass width, 3.00; and scanned mass range (*m*/*z*), 100–1600. The acquired MS/MS data were then compared with the in-house spectral libraries for the identification of the secondary metabolites. The identification was scored based on the similarity of fragmentation patterns between the acquired and the library spectra. The criteria we selected included MS2/MS3 fragment peak intensity ratios and isotope peak intensity ratios, among others. This acquisition scheme allowed the identification and characterization of not only the major (poly)phenolic compounds present in the studied extracts but also several low-level molecules [28,29,30].

### 2.11. Trace Metals Determination

All materials contacting the samples were soaked in dilute HNO_3_ (Merck, Darmstadt, Germany) and rinsed thoroughly with ultrapure water of 18.2 MΩ cm (Millipore, Bedford, MA, USA). Class A volumetric glassware was used for preparing all solutions required. For trace metals determination, samples of both plants and prepared decoctions were wet-digested by adding HNO_3_ 65% supra pure (Merck) and H_2_O_2_ 30% supra pure (Merck). Digestion was performed by a microwave digestion system (Anton Paar Multiwave GO Plus, Graz, Austria) and digested samples were subsequently diluted to a final volume of 25 mL [31]. The measurement of trace metals was carried out by Inductively Coupled Plasma Mass Spectrometry (ICP-MS), employing a Thermo Scientific ICAP Qc (Waltham, MA, USA) instrument, in a single collision cell mode, with kinetic energy determination (KED) using pure He. Matrix-induced signal suppressions and instrumental drift were corrected by internal standardization (45 Sc, 103 Rh).

Calculation of limits of detection (LODs) was performed by multiplying the standard deviation of seven replicate samples prepared at an approximately low concentration by 3.14 [32]. Calculated LODs in μg g^−1^ referring to dry weight were equal to 0.003 for Cd, 0.004 for Co and Ni, 0.02 for Cr, Cu and Mn, 0.05 for Fe, 0.008 for Pb and 0.04 for Zn. For quality assurance purposes, a procedural blank was included in samples’ analyses, in which no analytes were detected. For the verification of the accuracy and precision of the method, the certified reference material ERM^®^-CD281 (rye grass) was analyzed, with calculated metal recoveries ranging between 90 and 110%.

Metal extractability from the herb towards the aqueous infusion was calculated from their corresponding metal contents, while also considering the solid residue per cup of infusion and the amount of 3 g of the plant used for infusion preparation:

% Extraction Efficiency (EE) = 100 × (Metal content infusion × Solid residue per cup)/(Metal content plant tissue × 3)

## 3. Results and Discussion

### 3.1. Secondary Metabolites

Table 1 represents the analytical data for total phenolic, total flavonoid, total hydroxycinnamate, total flavonol, total anthocyanin and total terpenic contents of the decoctions and methanolic and aqueous-glycerolic extracts of *A. arborescens* and *A. inculta*. It is well-known that the three fundamental classes of bioactive compounds of *Artemisia* are flavonoids, phenolic acids and terpenes [33]. In total, in our samples, aqueous-glycerolic extracts of both species had higher levels of the above secondary metabolites compared to decoctions and methanolic extracts. The content of total (poly)phenolic compounds of the aqueous-glycerolic extracts of *A. arborescens* and *A. inculta* has been investigated before, being dependent on the concentration of glycerol and the liquid-to-solid ratio [19]. Different methanol, ethanol and acetonitrile extracts of A. absinthium contained TPC ranging from 659 to 1033 mg gallic acid equivalents/100 g dm (dry matter), and TFC ranging from 259 to 392 mg catechin equivalents/100 g dm [34]. Singh et al. [35] reported that TPC and TFC were higher in ethanolic extracts of *A.* absinthium compared to aqueous and chloroform extracts, suggesting the organic solvent (ethanol) is ideal to extract bioactive phenolic compounds. In our study, the aqueous-glycerol extract exhibited the greater potential to possess more polyphenols and terpenes. 

In the study by Bourgou et al. [36], ethyl acetate fractions of *A. herba-alba* showed higher quantity of TPC (87.5 mg gallic acid equivalents /100 g dm) and TFC (96.5 mg QE/g dm) compared to the water fraction (TPC = 40 mg gallic acid equivalents/100 g dw, TFC = 60.6 mg QE/g dm).

### 3.2. Antioxidant Properties

Τhe aqueous-glycerol extract exhibits greater antioxidant potential compared to decoctions and methanolic extracts, as shown in Table 2. More specifically, scavenging/antiradical activity, as assessed by DPPH assay, antioxidant power as assessed by FRAP assay and inhibition of copper-induced lipid oxidation in total serum were higher in the aqueous-glycerol extract. Several studies have proven the antioxidant properties of leaf extracts and essential oil of *Artemisia* species and have linked these effects with their components [37,38,39,40].

### 3.3. GC-MS Profiling

GC-MS analysis provided the composition of the predominant phenolic and terpenoid compounds in the studied *Artemisia* preparations (Table 3). In total, 22 compounds were identified. Most phenolic compounds were detected at higher levels or appeared only in the methanolic extract, whereas terpenoids were detected at higher levels or presented only in the aqueous-glycerol extract. Comparing the two species, a great variation was observed, with some phenolic compounds being higher in the methanolic extract or the aqueous-glycerol extract of *A. arborescens* or *A. inculta*, and some others being higher in the decoctions of both species. Ursolic acid was higher in *A. inculta* and in aqueous-glycerol extracts compared to methanolic ones, and oleanolic acid was higher in aqueous-glycerol extracts compared to methanolic ones, but higher in *A. inculta* compared to *A. arborescens* only in the aqueous-glycerol extract. Ursolic and oleanolic acids have been isolated from *A. indica*, showing modulatory effects on *γ*-Aminobutyric acid (GABA-A) receptors, demonstrating anxiolytic activity in mouse models, with no signs of acute toxicity [41]. Additionally, ursolic acid isolated from the methanolic extracts of *A. capillaris* inhibited the growth of both susceptible and resistant strains of Mycobacterium tuberculosis, exhibiting promising results against tuberculosis [42]. It is noteworthy that erythrodiol and uvaol, well-known for their antioxidant and anti-inflammatory activities [43,44], were identified only in the aqueous-glycerol extracts. To the best of our knowledge, this is the first time these two terpenoids are identified in an *Artemisia* extract.

### 3.4. HPLC-MS Profiling in Hydro-Glycolic Extracts

Based on the results reported for the majority of spectrophotometric assays, the glycerolic extracts were further analyzed by applying LC-MS as it is more suitable for the determination of a wider range of polar and semi-polar compounds, which are often present in plant glycerolic extracts [45]. Under this perspective, we proceeded with a targeted LC-MS method to separate and detect individual (poly)phenolic compounds in the glycerolic extracts of *Artemisia* in order to further investigate the phytochemical profile of the studied glycerol extracts. Tandem mass spectrometry (MS/MS) and built-in MRM spectral libraries were employed to confirm the identity of the compounds.

Table 4 demonstrates the phenolic compounds identified in the aqueous-glycerolic extracts of *A. arborescens* and *A. inculta*. Chlorogenic acid, isorhamnetin, kaempferol-3-O-glucoside and kaempferol-3-O-rutinoside were common in the composition of extracts from both *Artemisia* species. Slimestad et al. [46] identified chlorogenic acid, the ester of caffeic and quinic acid, in both leaves and stalks of *A. annua*, while the antimicrobial potency of extracts from wormwood (*A. gmelinii*) against Gram-positive bacteria and Candida spp. was partially attributed to chlorogenic acid, which dominated the ethanolic preparation from the aerial parts of the plant [47]. High yields of chlorogenic acid from sweet wormwood (*A. annua*) and tarragon (*A. dracunculus*) were obtained in ethanolic fractions that elicited strong radical-scavenging activity [48]. Further, isorhamnetin, naturally contained in Hippophaerhamnoides and Ginkgo biloba [49], was also detected in *A. Annua* [50]. This O-methylated flavonol can protect against atherosclerosis [51], also displaying significant anti-tumor activity [52]. Several kaempferol glycosides were also reported in infusions from the aerial parts of *A. copa Phil*. [53] while kaempferol-3-O-glucoside (astragalin) and its aglycone flavonol, i.e., 3,4′,5,7-tetrahydroxyflavone, were predominant in *A. annua* [50]. Kaempferol is a common dietary flavonoid that exhibits antioxidant and anti-inflammatory effects [54].

Notably, the phenolic profile, as determined by LC-MS, greatly differentiated between the two *Artemisia* species. A total of 14 phenolic targets were present in *A. arborescens* glycerol extract, while dihydrokaempferol 3-O-glucoside, procyanidin B2, hexose ester of protocatechuic acid, p-hydroxybenzoic acid and quercetin-O-xyloside were among others that were not detected in *A. inculta*. Other researchers have reported simple phenolic compounds and flavonoids found in *A. arborescens* solvent fractions, namely p-coumaric and caffeic acids, chrysosplenol-D, casticin, eupatin, cirsilineol, chrysosplenetin and artemetin [55].

According to our results reported in Table 4, *A. inculta* glycerolic fraction investigation hit 12 positive results corresponding to MRM mass spectra of (poly)phenolic compounds included in the in-house library, such as caffeic acid hexoside, dihydrokaempferol-3-O-rhamnoside, ellagic acid, quercetin-3-O-glucoside and syringetin-hexoside. There is rather limited published data so far on the (poly)phenolic profile of *A. inculta*. However, a study by Younsi et al. [56] indicated chlorogenic acid and 1,4 dicaffeoylquinic acid as the major phenolic constituents in a methanolic extract from *A. inculta* leaves and flowers, while apigenin-6-C-glycosyl flavonoids and caffeoylquinic acids were also present. A recent study revealed that caffeic acids and C-glycosyl flavonoids, such as myricetin, prevail in the (poly)phenolic composition of different extracts from the specific *Artemisia* species [57]. Furthermore, Mohammed et al. [58] reported on significant levels of hydroquinone and 4-hydroxybenzoic acid in *A. inculta* extracts demonstrating antibacterial activity. To the best of our knowledge, ellagic acid, a hydroxybenzoic acid dimer, has not been previously reported in *A. inculta*; however, it was a main phenol in *A. aucheri* [59], while an ellagic acid derivative was detected in *A. argentea L’ Hér* alcoholic extract [40]. Ellagic acid is abundant in various fruits such as pomegranate, strawberry, raspberry and blackberry. It is also found in nuts such as walnuts, certain trees such as oak and birch and some medicinal plants and herbs, including Terminalia chebula and Eucalyptus globulus [8]. Ellagic acid is considered a prominent bioactive compound due to its potential health-promoting properties. It has been shown to possess several properties such as antioxidant [60], anti-inflammatory [61] and cardioprotective activities [62].

### 3.5. Trace Metals

Levels of detected trace metals in *A. arborescens* and *A. inculta* samples are presented in Table 5. The samples differed in terms of their Cd, Co, Cr, Mn, Ni and Pb contents, with *A. arborescens* demonstrating higher concentrations in both the herbal tissue and infusion samples. The concentrations of Cu, Fe and Zn were comparable between the two species. Among the trace metals examined, Fe, Mn and Zn were present at higher levels in both the herbal tissues and corresponding infusions of *A. arborescens* and *A. inculta* samples. Fe, which is an essential element, represents the principal component in several enzymes and proteins and plays a crucial role in the transportation of oxygen to the tissues of the human body through hemoglobin [63], varied between 44.8 and 228 μg g^−1^. Mn, which is also classified among essential elements, participates in enzymes and contributes to oxidative stress response [64], bone formation and metabolism of amino acids, cholesterol and carbohydrates [65], varied between 27.8 and 101 μg g^−1^. Zn, which enhances body immunity and protection against several diseases, maintaining a crucial role in many enzymes and participating in metabolic reactions [66], was measured from 33.8 to 56.1 μg g^−1^.

Comparatively lower concentrations were measured for Ni (1.47–41.9 μg g^−1^) and Cu (9.38–21.1 μg g^−1^), which, although essential, may exhibit a toxic impact when detected at elevated concentrations. Despite the relatively limited data available, a beneficial role of Ni in physiological processes of animal species has been demonstrated, together with potential carcinogenic effects accompanying exposure to nickel compounds [67]. Cr concentrations measured in *Artemisia* tissue and infusion samples analyzed herein were equal to 0.781–2.42 μg g^−1^. Even lower levels were detected for Co (0.117–0.554 μg g^−1^), which is closely associated with the physiological role of vitamin B12 in the production and maintenance of red blood cells. 

A relatively low content of the toxic elements Cd (0.059–0.621 μg g^−1^) and Pb (0.174–0.676 μg g^−1^) was determined in *Artemisia* tissue and infusion samples. Classified by the International Agency for Research on Cancer (IARC) as “carcinogenic to humans” [68] and ranked by the EU in category 1 [69], Cd has been characterized as responsible for renal tubular dysfunction, bone fragility and reproductive disorders following prolonged oral exposure. Concerning Pb, its well-demonstrated toxicity threatens both young children, with the central nervous system representing the target organ, as well as adults with the manifestation of chronic kidney disease and cardiovascular dysfunctions. The maximum permissible levels in raw plant materials, set at 0.3 μg g^−1^ for Cd and 10 μg g^−1^ for Pb by the World Health Organization [70], were exceeded only in the case of Cd measured in the *A. arborescens* tissue sample (0.621 μg g^−1^).

Values detected in *A. arborescens* are similar to those reported for various other *Artemisia* species (in μg g^−1^ per dry weight of herb) for Cd (0.05–0.75), Cu (5.9–16.9), Fe (79.1–209.3), Mn (47.7–75.2) and Zn (35.2–58.6), whereas they were lower for Pb (1.25–2.08) [71]. Begaa et al. [72] reported similar values for Co (0.27–0.30) and Cr (0.74–1.50) for A. campestris and *A. herba-alba*, while reporting lower values for Zn (13–18) and higher values for Fe (617–631). Values comparable to these of the present work were recently presented by Ait Bouzid et al. [73] for *A. herba-alba* samples as follows: Cd (0.02 ± 0.01), Cu (6.6 ± 0.5), Fe (499 ± 40), Mn (80.3 ± 6.5), Pb (1.50 ± 0.03) and Zn (22.5 ± 1.8). As regards other herbal species consumed in the form of infusions, comparable levels (in μg g^−1^ per dry weight of herb) of Cd (0.01–0.39), Cr (0.27–2.45) and Ni (2.70–13.41) as well as significantly higher levels of Cu (7.73–63.71) and Pb (0.48–10.57) were detected in a Chinese tea [74]. Similar results (in μg g^−1^) for Cd (0.16–0.68), Cu (4.19–9.49), Fe (79.4–522) and Pb (0.73–1.51) were reported by Kalny et al. [75] for *Taraxacum officinale* (dandelion), *Betula* sp. (birch) and *Crataegus* sp. (hawthorn), commonly used for tea preparation.

Herbal infusions are taken orally and ingested in our digestive system. The element fraction actually retained in the human body following consumption is determined by the levels of elements extracted in the infusion. The extraction efficiency of trace elements is further dependent on the plant species, the organic matrix composition of the infusion prepared and element incorporation therein, in the form of either different covalent species or coordination complexes. In addition to the trace elements content of the initial herbal tissue, the corresponding infusion provides, hence, valuable information [76]. Although the species of *Artemisia* examined differed in the extractability order of trace elements transferred from the herbal tissue towards the infusion, Cu was significantly extracted in both cases (55.6 and 41.3% for *A. arborescens* and *A. inculta*, respectively) followed by Co (59.7 and 35.5%), Zn (29.8 and 42.4%), Cr (14.8 and 35.5%), Mn (36.6 and 26.0%), Ni (29.1 and 36.5%) and Pb (14.0 and 46.6%) which migrated moderately, while Cd (13.0 and 24.6%) and Fe (6.2 and 7.0%) were poorly extracted (Table 5). According to Matsuura et al. [77] differences characterizing the extraction efficiencies of transition metals are difficult to explain, being related to their ionic and covalent features. 

To estimate the contribution of *Artemisia* infusion consumption to metal intake, a daily consumption of 2 cups (200 mL per cup) and a body weight of 65 kg were assumed. Metal concentrations expressed per cup for *A. arborescens* and *A. inculta* were, respectively, equal to 0.239 and 0.047 for Cd, 0.523 and 0.125 for Co, 1.06 and 0.833 for Cr, 15.5 and 12.6 for Cu, 42.3 and 36.7 for Fe, 95.6 and 22.3 for Mn, 19.5 and 1.60 for Ni, 0.281 and 0.242 for Pb and 49.6 and 42.9 for Zn. Potential intake of inadequate Fe levels might be responsible for a gradual reduction of Fe stores, further leading to Fe deficiency, a threat mainly to women. A Recommended Daily Intake (RDI) for Fe was set at 8–18 mg·day^−1^ [78]. Due to a lack of adequate data, no upper limit (UL) has been set for Mn so far, while its Adequate Intake (AI) was set at 5–5.5 mg·day^−1^ [79]. For Zn the RDI was set at 8–14 mg·day^−1^; however, due to the negative impact an excessive Zn intake might provoke, its UL has been set at 5–40 mg·day^−1^ [79]. For Fe, Mn and Zn, a 2-cup daily consumption of *Artemisia* infusions contributed to less than 3% of the corresponding lower bounds. Regarding the potentially toxic metals examined, a Tolerable Daily Intake (TDI) for Ni was recently established equal to 13 μg·kg^−1^ bw·day^−1^ [80] and due to lack of adequate evidence, EFSA [78] adopted a TDI of 300 μg kg^−1^ bw^·^day^−1^ for Cr. Based on the classification of Co(II) compounds as “possibly carcinogenic to humans” [81], a TDI equal to 1.6–8 μg·kg^−1^ bw·day^−1^ was set [82]. In all cases of Ni, Co and Cr, daily consumption contributed to an intake not exceeding 5%. A Provisional Tolerable Weekly Intake (PTWI) of 2.5 μg·kg^−1^ bw·week^−1^ has been set for Cd by EFSA [83], while, due to Pb toxicity, a PTWI has been set at 25 μg·kg^−1^ bw·week^−1^ in 1986 by JECFA. The latter is a health guidance value that, however, has been withdrawn and not replaced so far [84]. In both cases, *Artemisia* infusions contributed at a percentage not exceeding 2%.

## 4. Conclusions

The results of our study highlight the potential use of the investigated *Artemisia* species not only in the nutrition and food industry, but also in the development of dermo-cosmetic applications, as glycerol is well known for its ability to increase the transdermal delivery of active substances. The latter should be seen from the perspective of the increased consumer demand for plant-derived substances in cosmetology, as well as for more green and sustainable products in general.

## Figures and Tables

**Table 1:** Secondary metabolites detected in *A. arborescens* and *A. inculta* extracts.

	*A. arborescens*			*A. inculta*		
	Decoction	Methanolic	Aqueous-Glycerolic	Decoction	Methanolic	Aqueous-Glycerolic
TPC (mg CAE·g^−1^ dm)	8.29 ± 0.27 ^d^	7.3 ± 1.1 ^d^	32.82 ± 0.50 ^b^	9.74 ± 0.15 ^c^	10.80 ± 0.90 ^c^	36.0 ± 2.5 ^a^
TFC (mg CE·g^−1^ dm)	6.05 ± 0.17 ^c^	4.48 ± 0.37 ^d^	19.57 ± 0.76 ^a^	6.04 ± 0.72 ^c^	7.0 ± 1.3 ^c^	16.77 ± 0.36 ^b^
THCC (mg CAE·g^−1^ dm)	0.0985 ± 0.0035 ^e^	0.095 ± 0.017 ^d,e^	0.310 ± 0.010 ^b^	0.1117 ± 0.0012 ^d^	0.1257 ± 0.0050 ^c^	0.338 ± 0.013 ^a^
TFnolC (mg QE·g^−1^ dm)	0.0859 ± 0.0025 ^e^	0.100 ± 0.016 ^d,e^	0.293 ± 0.015 ^b^	0.0988 ± 0.0015 ^d^	0.1383 ± 0.0048 ^c^	0.370 ± 0.013 ^a^
TAC (μg CNE·g^−1^ dm)	0.651 ± 0.032 ^d^	1.424 ± 0.028 ^b^	2.680 ± 0.082 ^a^	0.462 ± 0.011 ^e^	0.764 ± 0.058 ^c^	2.61 ± 0.14 ^a^
TTC (mg UAE·g^−1^ dm)	0.229 ± 0.029 ^d^	1.859 ± 0.160 ^a^	0.374 ± 0.021 ^c^	0.215 ± 0.021 ^d^	1.857 ± 0.346 ^a^	0.438 ± 0.024 ^b^

Total phenolic content was expressed as caffeic acid equivalents (CAE), total flavonoid content as catechin equivalents (CE), total hydroxycinnamate content as caffeic acid equivalents (CAE), total flavonol content as quercetin equivalents (QE), total anthocyanin content as cyanidin equivalents (CNE), and total terpenic content as ursolic acid equivalents (UAE) on a dry material basis for the decoctions, methanolic (MeOH), and glycerolic extracts of *A. arborescens* and *A. inculta*. Values are presented as mean (±standard deviation) (*n* = 3). TPC: total phenolic content, TFC: total flavonoid content, THCC: total hydroxycinnamate content, TFnolC: total flavonol content, TAC: total anthocyanin content, TTC: total terpenic content, dm: dry matter. ^a–e^ Means per row denoted by a common superscript letter are not significantly different according to Tukey’s test at 5% level of significance.

**Table 2:** Antioxidant potential of *A. arborescens* and *A. inculta* extracts.

	*A. arborescens*			*A. inculta*		
	Decoction	Methanolic	Aqueous-Glycerolic	Decoction	Methanolic	Aqueous-Glycerolic
Antiradical activity (mg TE·g^−1^ dm)	5.18 ± 0.50 ^e^	7.524 ± 0.039 ^d^	30.9 ± 1.2 ^a^	5.68 ± 0.21 ^e^	8.23 ± 0.40 ^c^	27.8 ± 1.1 ^b^
FRAP (mg AAE·g^−1^ dm)	3.45 ± 0.14 ^e^	5.36 ± 0.44 ^c^	21.77 ± 0.70 ^a^	2.92 ± 0.25 ^e^	4.31 ± 0.47 ^d^	18.19 ± 0.27 ^b^
TSO (sec)	1107.7 ± 8.1 ^f^	627.8 ± 366.3 ^a^	2007.25 ± 171.25 ^f^	2120 ± 493.8 ^b^	1400.3 ± 377.2 ^f^	3850.25 ± 320.25 ^c^

^a–f^ Means per row denoted by a common superscript letter are not significantly different according to Tukey’s test at 5% level of significance. Values are presented as mean (±standard deviation) (*n* = 3). TE: Trolox equivalents, FRAP: Ferric Reducing/Antioxidant Power, TSO: total serum oxidizability.

**Table 3:** Composition of *A. arborescens* and *A. inculta* extracts samples assessed by GC-MS (expressed as μg per g of dry material).

Phenolic Compounds	Molecular Formula	*A. arborescens*			*A. inculta*		
		Decoction	Methanolic	Aqueous-Glycerolic	Decoction	Methanolic	Aqueous-Glycerolic
Caffeic acid	C_9_H_8_O_4_	94.6 ± 7.5 ^d^	121.9 ± 9.0 ^c^	39.1 ± 4.5 ^e^	382 ± 12 ^a^	289.6 ± 6.7 ^b^	19.1 ± 3.0 ^f^
Chlorogenic acid	C_16_H_18_O_9_	537.1 ± 8.9 ^e^	5754 ± 70 ^a^	1669.2 ± 1.3 ^d^	2332 ± 179 ^c^	5052 ± 56 ^b^	285 ± 19 ^f^
Chrysin	C_15_H_10_O_4_	nd	3.89 ± 0.05 ^b^	nd	nd	6.51 ± 0.13 ^a^	nd
p-Coumaric acid	C_9_H_8_O_3_	1.52 ± 0.13 ^d^	2.97 ± 0.32 ^c^	nd	37.78 ± 0.26 ^b^	43.2 ± 3.5 ^a^	nd
Ferulic acid	C_10_H_10_O_4_	16.26 ± 0.59 ^b^	2.54 ± 0.16 ^d^	1.64 ± 0.05 ^e^	25.3 ± 1.4 ^a^	16.8 ± 1.4 ^b^	3.94 ± 0.06 ^c^
Gallic acid	C_7_H_6_O_5_	nd	0.94 ± 0.02 ^a^	nd	nd	0.67 ± 0.06 ^a^	nd
p-Hydroxybenzoic acid	C_7_H_6_O_3_	7.89 ± 0.16 ^c^	1.16 ± 0.10 ^e^	3.82 ± 0.46 ^d^	45.4 ± 2.6 ^a^	nd	23.99 ± 0.42 ^b^
p-Hydroxyphenylacetic acid	C_8_H_8_O_3_	nd	0.39 ± 0.05 ^b^	nd	nd	0.44 ± 0.03 ^b^	5.17 ± 0.68 ^a^
Kaempferol	C_15_H_10_O_6_	nd	1.15 ± 0.11 ^b^	nd	nd	3.19 ± 0.18 ^a^	nd
Naringenin	C_15_H_12_O_5_	nd	3.07 ± 0.28 ^c^	nd	15.84 ± 0.25 ^b^	40.1 ± 2.7 ^a^	36.5 ± 2.4 ^a^
Phloretic acid	C_9_H_10_O_3_	nd	nd	1.70 ± 0.09 ^a^	nd	0.49 ± 0.01 ^b^	1.02 ± 0.02 ^c^
Protocatechuic acid	C_7_H_6_O_4_	8.73 ± 0.80 ^b^	6.44 ± 0.33 ^b,c^	nd	21.2 ± 1.0 ^a^	5.51 ± 0.61 ^c^	nd
Quercetin	C_15_H_10_O_7_	nd	7.21 ± 0.31 ^c^	nd	14.93 ± 0.89 ^b^	23.04 ± 0.54 ^a^	nd
Resveratrol	C_14_H_12_O_3_	nd	0.36 ± 0.04 ^b^	1.62 ± 0.09 ^a^	nd	0.24 ± 0.03 ^b^	nd
Sinapic acid	C_11_H_12_O_5_	nd	n^d^	30.1 ± 3.0	nd	nd	nd
Syringic acid	C_9_H_10_O_5_	6.47 ± 0.51 ^b^	2.96 ± 0.29 ^c^	6.34 ± 0.47 ^b^	10.39 ± 0.26 ^a^	5.84 ± 0.37 ^b^	7.38 ± 0.13 ^b^
Tyrosol	C_8_H_10_O_2_	nd	0.05 ± 0.01 ^b^	nd	nd	0.14 ± 0.01 ^a^	nd
Vanillic acid	C_8_H_8_O_4_	6.66 ± 0.70 ^d^	2.20 ± 0.21 ^f^	3.06 ± 0.17 ^e^	21.1 ± 1.1 ^a^	13.96 ± 0.44 ^b^	9.18 ± 0.58 ^c^
Total Phenolic Compounds		679 ± 19 ^e^	5911 ± 81 ^a^	1756 ± 10 ^d^	2906 ± 199 ^c^	5502 ± 72 ^b^	392 ± 27 ^f^
Terpenoids							
Erythrodiol	C_30_H_50_O_2_	nd	nd	487.8 ± 14 ^a^	nd	nd	420 ± 30 ^a^
Oleanolic acid	C_30_H_48_O_3_	nd	8.54 ± 0.86 ^c^	242.6 ± 18 ^b^	nd	7.14 ± 0.68 ^c^	480 ± 45 ^a^
Ursolic acid	C_30_H_48_O_3_	nd	14.24 ± 0.93 ^c^	35.2 ± 5.0 ^b^	nd	15.58 ± 0.63 ^c^	82.4 ± 7.9 ^a^
Uvaol	C_30_H_50_O_2_	nd	nd	712.9 ± 18 ^a^	nd	nd	584 ± 42 ^b^
Total Terpenoids		nd	22.8 ± 1.8 ^b^	1478 ± 56 ^a^	nd	22.7 ± 1.3 ^b^	1568 ± 126 ^a^

nd: Not detected. ^a−f^ Means per row denoted by a common superscript letter are not significantly different according to Tukey’s test at 5% level of significance. Values are presented as mean (±standard deviation) (*n* = 3).

**Table 4:** Phenolic compounds identified in *Artemisia* spp. aqueous-glycerolic extracts with HPLC-ESI(–)-MS/MS(MRM) analysis.

Phenolic Compound	Molecular Formula	[M–H]—(*m*/*z*) ^1,2^	MS ^2^ Product Ions (*m*/*z*)	*A. arborescens*	*A. inculta*
Caffeic acid hexoside	C_15_H_18_O_9_	341.11	179, 161, 135		+
Chlorogenic acid	C_16_H_18_O_9_	353.15	217, 191	+	+
Dihydrokaempferol 3-O-glucoside	C_21_H_22_O_11_	449.09	287	+	
Dihydrokaempferol-3-O-rhamnoside (Engeletin)	C_21_H_22_O_10_	433.00	269, 179, 151		+
Procyanidin B2	C_30_H_26_O_12_	577.24	425	+	
Ellagic acid	C_14_H_6_O_8_	301.06	301, 257, 229, 185		+
Ellagic acid-O-hexoside	C_20_H_16_O_13_	463.11	301, 300, 283, 257, 229		+
Gallic acid derivative	not defined	243.27	169, 225, 151, 139, 125	+	
Hexose ester of protocatechuic acid	C_13_H_15_O_9_	314.77	153	+	
p-Hydroxybenzoic acid	C_7_H_6_O_3_	137.06	93	+	
Isorhamnetin	C_16_H_12_O_7_	315.20	300, 301	+	+
Kaempferol-3-O-glucoside (Astragalin)	C_21_H_20_O_11_	447.24	285, 255, 327	+	+
Kaempferol-3-O-rutinoside (Nictoflorin)	C_27_H_30_O_15_	593.26	285	+	+
Phlorizin	C_21_H_24_O_10_	435.20	297, 273, 167	+	
Pyrogallol	C_6_H_6_O_3_	125.06	106, 97, 81	+	
Quercetin-3-O-glucuronide (Miquelianin)	C_21_H_18_O_13_	477.26	301	+	
Quercetin-3-O-glucoside	C_21_H_20_O_12_	463.19	301		+
Quercetin-O-xyloside	C_20_H_18_O_11_	433.19	301	+	
Syringaldehyde	C_9_H_10_O_4_	181.12	166		+
Syringetin-3-O-glucoside	C_23_H_24_O_13_	507.25	345		+
Syringetin-hexoside	C_23_H_24_O_13_	507.25	345, 327, 315		+
Valoneic acid bilactone	C_21_H_10_O_13_	469.04	425, 407	+	

^1^ Ions with relative abundance greater than 10% are shown; ^2^ [M–H]^–^: parent ion derived from molecular mass under negative electrospray ionization conditions; a positive identification for a phenolic compound in the glycerolic extracts is marked with the plus sign symbol (+).

**Table 5:** Levels of trace metals in dry herbal tissues and prepared infusions (μg g^−1^) of *A. arborescens* and *A. inculta* and extraction efficiency (% EE) of metals from the herb to the infusion.

	Cd	Co	Cr	Cu	Fe	Mn	Ni	Pb	Zn
***A. arborescens***									
Herbal tissue	0.621 ± 0.056	0.295 ± 0.027	2.42 ± 0.03	9.38 ± 1.02	228 ± 25	87.9 ± 10.0	22.6 ± 1.9	0.676 ± 0.056	56.1 ± 6.0
Infusion	0.254 ± 0.021	0.554 ± 0.049	1.12 ± 0.14	16.4 ± 1.88	44.8 ± 5.2	101 ± 11	41.9 ± 5.1	0.298 ± 0.031	52.6 ± 4.8
% EE	13.0	59.7	14.8	55.6	6.2	36.6	29.1	14.0	29.8
***A. inculta***									
Herbal tissue	0.064 ± 0.007	0.117 ± 0.011	0.781 ± 0.063	10.1 ± 0.9	175 ± 18	28.6 ± 3.4	1.47 ± 0.12	0.174 ± 0.016	33.8 ± 2.9
Infusion	0.059 ± 0.006	0.156 ± 0.013	1.04 ± 0.12	21.1 ± 1.9	45.8 ± 5.7	27.8 ± 2.2	4.77 ± 0.51	0.303 ± 0.036	
% EE	24.6	35.5	35.5	41.3	7.0	26.0	36.5	46.6	42.4

Cd: cadmium, Co: cobalt, Cr: chromium, Cu: copper, Fe: iron, Mn: manganese, Ni: nickel, Pb: lead, Zn: zinc.

## Data Availability

Data will be made available on request from the corresponding author.

## Supplementary Materials

The following supporting information can be downloaded at: https://www.mdpi.com/article/10.3390/life13061416/s1, Table S1: Target and qualifier ions for the trimethylsilyl ethers (TMS) of simple phenols, stilbenes, terpenic compounds, and the internal standard (IS).
