# Supplementary material for: Artemisia arborescens and Artemisia inculta from Crete; Secondary Metabolites, Trace Metals and In Vitro Antioxidant Activities
Source: Life (Basel). 2023 Jun 19;13(6):1416. doi: 10.3390/life13061416 (PMC10304051; doi:10.3390/life13061416)
Supplement: Supplementary file 1 [file life-13-01416-s001.zip › life-2429637-supplementary.pdf]

Supplementary material related to the article

Artemisia arborescens and *Artemisia inculta* from Crete; secondary metabolites, trace metals and *in vitro* antioxidant activities

Table S1. Target and qualifier ions for the trimethylsilyl ethers (TMS) of simple phenols, stilbenes, terpenic compounds, and the internal standard(IS).

Compound	Rt (min)	Molecular Formula	Type	Target ion	Qualifier ions
Phenolic compounds					
3-(4-hydroxyphenyl)-1-propanol (IS)	22.20	$C_9H_{12}O_2$	I.S. (Phenol)	206	191, 179
Caffeic acid	39.48	$C_9H_8O_4$	Phenol	396	219, 381
Chlorogenic acid	48.78	$C_{16}H_{18}O_9$	Phenol	345	307, 324
Chrysin	45.44	$C_{15}H_{10}O_4$	Phenol	383	355, 474
p-Coumaric acid	35.38	$C_9H_8O_3$	Phenol	308	293, 219
Ferulic acid	38.77	$C_{10}H_{10}O_4$	Phenol	338	323, 308
Gallic acid	36.64	$C_7H_6O_5$	Phenol	281	458, 443
p-Hydroxybenzoic acid	19.80	$C_7H_6O_3$	Phenol	267	223, 193
p-Hydroxyphenylacetic acid	20.50	$C_8H_8O_3$	Phenol	252	296, 281
Kaempferol	48.15	$C_{15}H_{10}O_6$	Phenol	559	560
Naringenin	46.28	$C_{15}H_{12}O_5$	Phenol	473	296
Phloretic acid	26.11	$C_9H_{10}O_3$	Phenol	192	310
Protocatechuic acid	30.70	$C_7H_6O_4$	Phenol	193	355, 370
Quercetin	49.44	$C_{15}H_{10}O_7$	Phenol	647	575
Resveratrol	45.03	$C_{14}H_{12}O_3$	Stilbene	444	445, 443
Sinapic acid	40.54	$C_{11}H_{12}O_5$	Phenol	368	353, 338
Syringic acid	34.77	$C_9H_{10}O_5$	Phenol	327	342, 312
Tyrosol	18.31	$C_8H_{10}O_2$	Phenol	179	267, 282
Vanillic acid	26.53	$C_8H_8O_4$	Phenol	297	267, 312
Terpenic compounds					
Erythrodiol	55.47	$C_{30}H_{50}O_2$	Terpenic alcohol	216	203, 189
Uvaol	56.22	$C_{30}H_{50}O_2$	Terpenic alcohol	203	216, 188
Oleanolic acid	55.86	$C_{30}H_{48}O_3$	Terpenic acid	203	202, 482
Ursolic acid	56.94	$C_{30}H_{48}O_3$	Terpenic acid	203	202, 482

A selective ion monitoring (SIM) GC–MS method was applied for the detection and identification of the trimethylsilyl ethers (TMS) of seventeen phenolic compounds, one stilbene, two terpenic alcohols and two terpenic acids based on the ±0.05 RT presence of target and qualifier ions of pure commercial standards at the predetermined ratios. Quantification was achieved by constructing reference curves for each compound and employing 3-(4-hydroxyphenyl)-1-propanol as internal standard.
